# Single-cell RNA-sequencing of peripheral blood mononuclear cells reveals the transcriptome profile of *Microtus fortis* immune cells during the early phase of infection with *Schistosoma japonicum*

**DOI:** 10.3389/fcimb.2025.1739541

**Published:** 2026-01-27

**Authors:** Nouhoum Dibo, Zhijun Zhou, Xianshu Liu, Zhuolin Li, Shukun Zhong, Jiajing Zhang, Huilan Wang, Bo Li, Xiaohui Yang, Yuehui Li, Xiang Wu, Shuaiqin Huang

**Affiliations:** 1 Department of Medical Parasitology, Xiangya School of Basic Medical Sciences, Central South University, Changsha, Hunan, China; 2 Hunan Key Laboratory of Immunology and Transmission Control of Schistosomiasis, Yueyang, Hunan, China; 3 Department of Laboratory Animals, Hunan Key Laboratory of Animal Models for Human Diseases, Central South University, Changsha, Hunan, China; 4 Schistosomiasis Control Institute of Hunan Province (The Third People’s Hospital of Hunan Province), Yueyang, Hunan, China; 5 Regional Collaboration Centre for Schistosomiasis Control Technology in Lake Regions, Yueyang, Hunan, China; 6 Nursing Department, The Third Xiangya Hospital of Central South University, Changsha, Hunan, China; 7 Department of Biomedical Information, School of Life Sciences, Central South University, Changsha, Hunan, China

**Keywords:** *Microtus fortis*, natural resistance, peripheral blood mononuclear cells, *S. japonicum*, single-cell RNA sequencing

## Abstract

**Introduction:**

The reed vole, *Microtus fortis*, is the only known natural non-permissive mammalian host of *Schistosoma japonicum*. However, the molecular mechanisms underlying this resistance have not been fully understood.

**Methods:**

We performed single-cell RNA-seq to investigate the peripheral blood mononuclear cells (PBMCs) responses to *S. japonicum* in *M. fortis* and the susceptible host, Kunming mice. The samples were collected from uninfected animals (control group) and infected animals at 10 dpi.

**Results:**

The major cell types identified in the PBMCs of the two species were monocytes, dendritic cells (DCs), T cells, NK cells, B cells, and erythrocytes. We observed that the population of monocytes decreased considerably in the bloodstream after infection in both *M. fortis* and Kunming mice. However, differential gene expression analysis revealed that Cxcl9 was upregulated in *M. fortis* monocytes after infection, while it was not detected as a DEG in Kunming mice. In addition, we observed that infection induced the upregulation of IL2 and IL4 in *M. fortis* CD4+ T cells, and the expansion of the Th2 cell population. Regarding B cells, we did not observe any significant alteration among *M. fortis* B cell subpopulations after infection compared to the control. However, DEG analysis revealed that Igha, Ighg1, and Ighg3 were upregulated in *M. fortis* antibody secreting cells (ASCs) but not in Kunming mice.

**Discussion:**

Together, our results suggest that both the innate and adaptive immune responses were activated in the peripheral blood of *M. fortis* at 10 dpi, while their activation was not obvious in Kunming mice at the same moment.

## 1 Introduction

Schistosomiasis, caused by the blood flukes of the genus *Schistosoma*, is a neglected tropical disease prevalent in tropical and subtropical regions. Humans become infected when the cercaria form of the parasite penetrates the skin during contact with infected water, mostly in rural communities lacking access to potable water and adequate sanitation (LoVerde, 2019). Although great efforts have been made in schistosomiasis control, its elimination as a public health problem remains a daunting challenge in many countries due to the insurmountable side effects and the potential drug resistance risk of the only commercially available antischistosomal drug, praziquantel (PZQ), and the lack of an effective vaccine to prevent reinfection (Molehin et al., 2022).

An alternative route for vaccine development and new therapy discovery is to elucidate the cellular and molecular basis of protective immunity displayed by animal models (Amaral et al., 2021). *Microtus fortis*, commonly known as the reed vole, is a unique mammal in which *S. japonicum* cannot complete its development. After skin penetration, the worm’s larvae migrate to the lungs of *M. fortis* within 2 days and then to the liver within 4 days, where their development is inhibited at 12 dpi, and the immature worms die by 3 weeks post-infection without causing significant damage (Li et al., 2000) (He et al., 2001). Both experimental and epidemiological studies strongly suggest that *M. fortis* is naturally resistant to *S. japonicum*, and this resistance cannot be affected by the environment (wild and laboratory-bred) (He et al., 1999b) (Shen et al., 2020).

The PBMCs, which include T lymphocytes, B lymphocytes, natural killer (NK) cells, and mononuclear phagocytes (MPs), allow researchers to analyze both innate and adaptive immune responses within a single sample (Khan and Kaihara, 2019). As these cells circulate throughout the body, their dynamics and transcriptomic profiles can provide insight into the host’s overall, system-wide immune engagement with the invading pathogens (Wang et al., 2025) (Hertaeg et al., 2025). They detect pathogen antigens through pattern recognition receptors (PRRs), transmitting signals into the cell to activate the expression of pro-inflammatory cytokines and chemokines, which regulate the host immune response (Derbois et al., 2023). Therefore, PBMCs are influential biological sensors of infection, making them suitable for studying immune responses against pathogenic infection (Zilionis et al., 2019). Previous studies have suggested that serum and lymphocytes from *M. fortis* exhibit *Schistosoma*-killing effects both *in vivo* and *in vitro* (He et al., 1999a) (Hu et al., 2012). It has also been reported that immunodeficient mice receiving a bone marrow transplant (BMT) from *M. fortis*, which increased total B and T lymphocytes, exhibited inhibition of *S. japonicum* development (Hu et al., 2014a). However, the transcriptional program of the immune cells has not been investigated. Single-cell next-generation sequencing is a powerful method that can analyze the host’s immune response at the single-cell level, providing a more efficient tool for deciphering the host’s immune responses (Wang et al., 2023). In this study, we compared the immune characteristics of *M. fortis* with those of the highly susceptible host Kunming mice using scRNA-Seq. The study enabled us to decipher the heterogeneity of *M. fortis* PBMCs and provide detailed information on the transcriptome programs of the different cell types. It will significantly contribute to understanding the molecular basis of *M. fortis* resistance to *S. japonicum* infection, and also lay the foundation for identifying vaccine candidates and novel drug targets against schistosome infection.

## 2 Materials and methods

### 2.1 Ethics statement

All the experimental procedures involved were performed strictly in accordance with the protocols (code 2021 KT-53) approved by the Ethics Committee of Xiangya School of Basic Medical Sciences, Central South University, Changsha, China. The protocols of housing, breeding, and care of the animals followed the ethical requirements of the government of China.

### 2.2 Animal infection and sample collection

Six *M. fortis* and six Kunming mice were used in this study. The Chinese mainland strain of *S. japonicum*, maintained by serial passage through *O. hupensis* snails and inbred Chinese Kunming mice in our laboratory, was used to infect the animals. For each species, three animals were infected with 100 cercariae, and three others were kept as a control group. The animals with free access to food and water were housed under specific-pathogen-free conditions at the experimental animal center. The peripheral blood was collected from uninfected and infected animals at 10 dpi. For sequencing cost reduction and processing a larger number of cells, we pooled the biological replicates to perform scRNA sequencing.

### 2.3 Single-cell isolation and preparation

The PBMCs were isolated by density gradient centrifugation using Ficoll-Paque Plus medium (GE Healthcare) and washed with Ca/Mg-free PBS. 2 mL GEXSCOPE^®^ red blood cell lysis bufier (RCLB, Singleron) was added at 25 °C for 10 min to remove the red blood cells. The solution was then centrifuged at 500 g for 5 min and suspended in PBS. The samples were centrifuged at 400g for 5 min at 4 °C, and the supernatant was discarded. After removing red blood cells, the PBMCs were resuspended in PBS and centrifuged at 400 g for 10 min at 4 °C to collect the cells after discarding the supernatant. Finally, the samples were stained with Trypan Blue, and the cell viability was evaluated microscopically.

### 2.4 Library preparation and Illumina sequencing

Single-cell suspensions (2×10^5^ cells/mL) with PBS (HyClone) were loaded onto a microwell chip using the Singleron Matrix^®^ Single Cell Processing System. Barcoding Beads were subsequently collected from the microwell chip, followed by reverse transcription of the mRNA captured by the barcoding beads to obtain cDNA and PCR amplification. The amplified cDNA is then fragmented and ligated with sequencing adapters. The scRNA-seq libraries were constructed according to the protocol of the GEXSCOPE^®^ Single Cell RNA Library Kits (Singleton). Individual libraries were diluted to 4 nM, pooled, and sequenced on Illumina Novaseq 6000 with 150 bp paired-end reads.

### 2.5 Single-cell data analysis

Raw reads from scRNA-seq were processed to generate a gene expression matrix using CeleScope (https://github.com/singleron-RD/CeleScope) v1.9.0 pipeline. Briefly, raw reads were firstly processed with CeleScope to remove low-quality reads, trim poly-A tail, and adapter sequences with Cutadapt v1.17 (Martin, 2011). STAR v2.6.1a was used to map the reads from the two species to the mouse reference genome GRCm38 (Ensembl version 92 annotation) (Dobin et al., 2013), because the current annotated reference genome of *M. fortis* (M_Fortis_MF-2015_v1) available in the National Center for Biotechnology Information (NCBI) is incomplete (Li et al., 2020). Each cell’s UMI and gene counts were acquired with feature Counts v2.0.1 software to generate expression matrix files for subsequent analysis (Liao et al., 2014).

For each sample dataset, we filtered the expression matrix by the following criteria: 1) cells with a gene count less than 200 were excluded; 2) cells with a top 2% UMI count were excluded; 3) cells with mitochondrial content > 20% were excluded; 4) genes expressed in less than 5 cells were excluded. The raw count matrix was normalized by total counts per cell and logarithmically transformed into a normalized data matrix. Seurat was used to cluster the merged object into subsets of cells. This workflow includes finding variable genes, running principal component analysis on variable genes, and running Uniform Manifold Approximation and Projection on Principal Components (UMAP) (Mangiola et al., 2021). In the first round of cells clustering (pre-clustering), resolution = 0.3 was used to identify major cell types, and resolution = 0.2 was used for the second round to identify mononuclear phagocytes (MPs), T and B cell subsets. Cell cluster identity was determined by finding DE genes for each cluster using Seurat’s FindAllMarkers () function and by comparing those markers to known cell-type-specific genes in the SynEcoSys database, combined with knowledge from the literature (Zhang et al., 2023) (Zhang et al., 2023). Differential gene expression analysis between conditions was performed using Seurat’s ‘findmarkers’ function based on the Wilcox likelihood-ratio test with default parameters. The genes with an avg_log2FC value greater than 0.25 and adjusted p-value less than 0.05 were considered differentially expressed genes (DEGs).

### 2.6 qPCR

We further collected PBMCs from uninfected and infected *M. fortis* at 10 dpi to perform qPCR for the validation of some potential candidate genes. Total RNA was extracted from *M. fortis* PBMCs using a Trizol reagent (Rio et al., 2010).

SYBR Green using Master Mix on a CFX96 Touch System (BioRad) was used to determine the expression level of IL2, IL4, Cxcl9, and Fcgrt (**Table 1**) in uninfected and infected *M. fortis*. Beta-actin (ACTB) was used as an internal control, and the relative expression was calculated using the 2^-△△Ct^ method. The data obtained for analysis were reported as mean values and standard deviation (SD), with 95% confidence intervals (CI); *P >*0.05 was considered statistically significant. All statistical analyses were conducted using GraphPad Prism 10.4.1.

**Table 1:** list of primer used for qPCR.

Gene	Forward/reverse	Primer sequence (5’->3’)
IL2	F	TTGGGAAACTGAAGGGCTCTG
	R	TCCACCACAGTTACCGTCTCA
IL4	F	GGTCACAGACAAAGGGACGC
	R	AAGTTCCCTCTCCGTGGTGT
Cxcl9	F	CCAGTGTGGGGTTCAAGGAAA
	R	GACCTGTAGGAGGGGATCGT
Fcgrt	F	AGGCCTGGGTTCCTAGTTCTG
	R	GACCCAGCCTTGCTGATTCT
ACTB	F	GCAGGAGTACGATGAGTCCG
	R	AAACGCAGCTCAGTCACAGT

## 3 Results

### 3.1 Major cell types identified in the PBMCs of the two species

**Figure 1:**
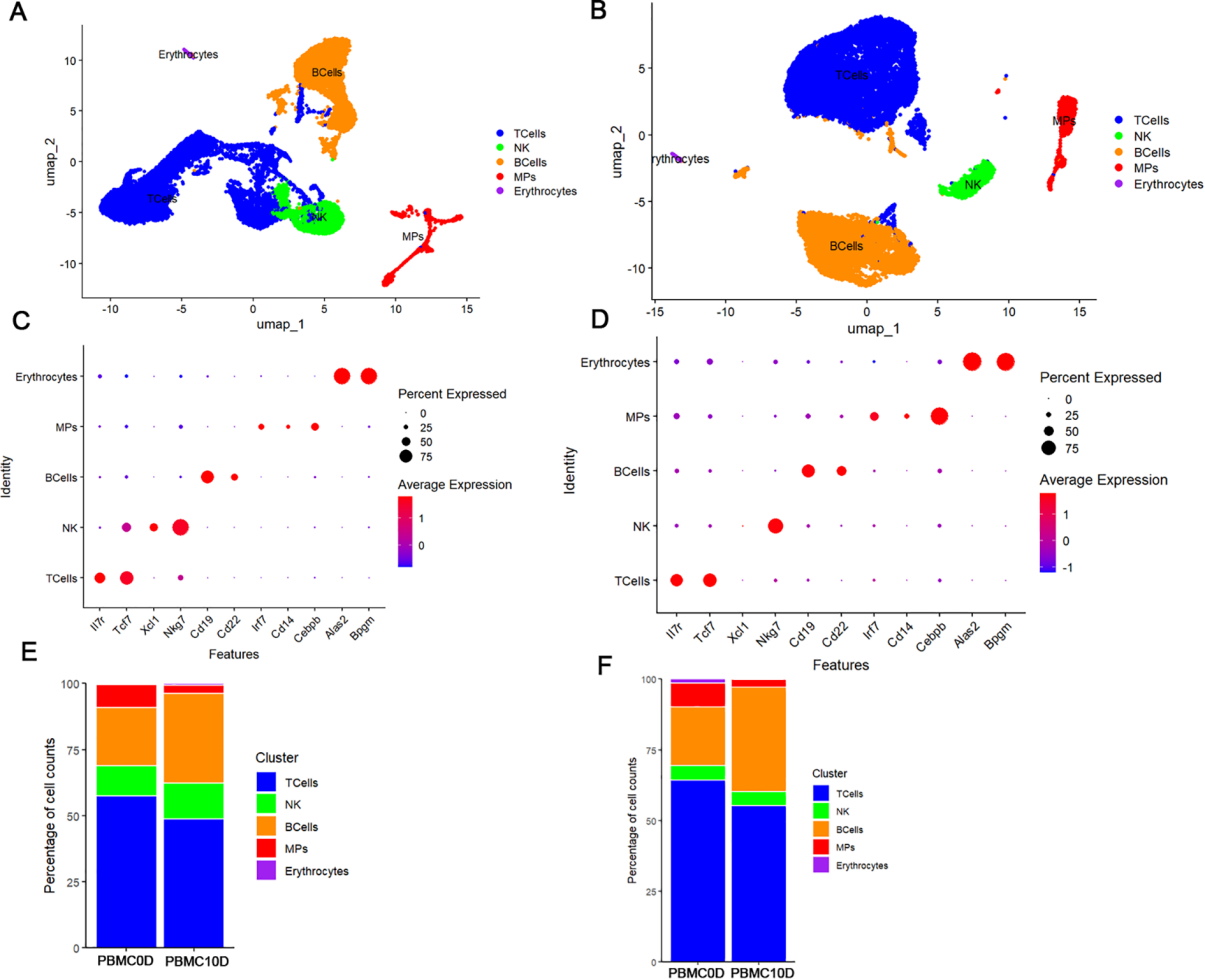
Clustering and cell type labeling of the PBMCs identified in the two species. **(A)** UMAP plot of *M. fortis* PBMCs. **(B)** UMAP plot of Kunming mouse PBMCs. **(C)** Dot plot of the top markers used to define *M. fortis* cell types. **(D)** Dot plot of the top markers used to define Kunming mouse cell types. The dot plot illustrates the average expression level and the percentage of cells expressing the marker genes across PBMC populations. **(E)** Percentage change in cell type across conditions in *M. fortis*. **(F)** Percentage change in cell type across conditions in Kunming mice. PBMC0D mean uninfected animal, PBMC10D mean infected animals of 10 dpi.

The total number of valid reads obtained in the different pools of samples varied from 349,110,435 to 412,975,949, and the mapping ratio varied from 70.68% to 80.92% (**Table 2**). After quality control, we recovered 20667 cells in *M. forti*s and 23147 cells in Kunming mice, respectively (**Table 2**). Based on the UMAP plot and the conserved marker genes, we grouped the PBMCs into T cells, natural killer (NK) cells, B cells, mononuclear phagocytes (MPs), and erythrocytes in both *M. fortis* and Kunming mice (**Figures 1A–D**). T cells were identified by the expression of Il7r and Tcf7, and NK cells were distinguished from T cells by the relatively high expression of Xcl1 and Nkg7 (**Figures 1C, D**). B cells were identified by the expression of Cd19 and Cd22, MPs by the expression of Irf7, Cd14, and Cebpb, and erythrocytes by the expression of Alas2 and Bpgm (**Figures 1C, D**). Cell type percentage comparison revealed that the percentage of T cells and MPs decreased in the blood of both *M. fortis* and Kunming mice at 10 dpi. In contrast, the percentage of B cells increased, suggesting that infection can induce dynamic changes in peripheral immune cell populations (**Figures 1E, F**).

**Table 2:** Quality control metrics.

id	Raw reads	Valid reads	Uniquely mapped reads	Multi-mapped reads	Number of cells	Total genes	Median genes per cell
MF-0D	375,169,016	349,110,435 (93.05%)	240,294,537 (71.13%)	50,513,859 (14.95%)	8043	16,123	1,558
MF-10D	372,800,999	344,277,132 (92.35%)	236,965,044 (70.68%)	49,309,739 (14.71%)	12624	16,427	1,516
KM-0D	390,777,764	360,391,044 (92.22%)	277,053,845 (80.92%)	40,778,327 (11.91%)	8062	24,355	15,085
KM-10D	454,584,306	412,975,949 (90.85%)	283,649,891 (73.98%)	39,806,830 (10.38%)	15085	24,746	931

### 3.2 Mononuclear phagocytes characterization

**Figure 2:**
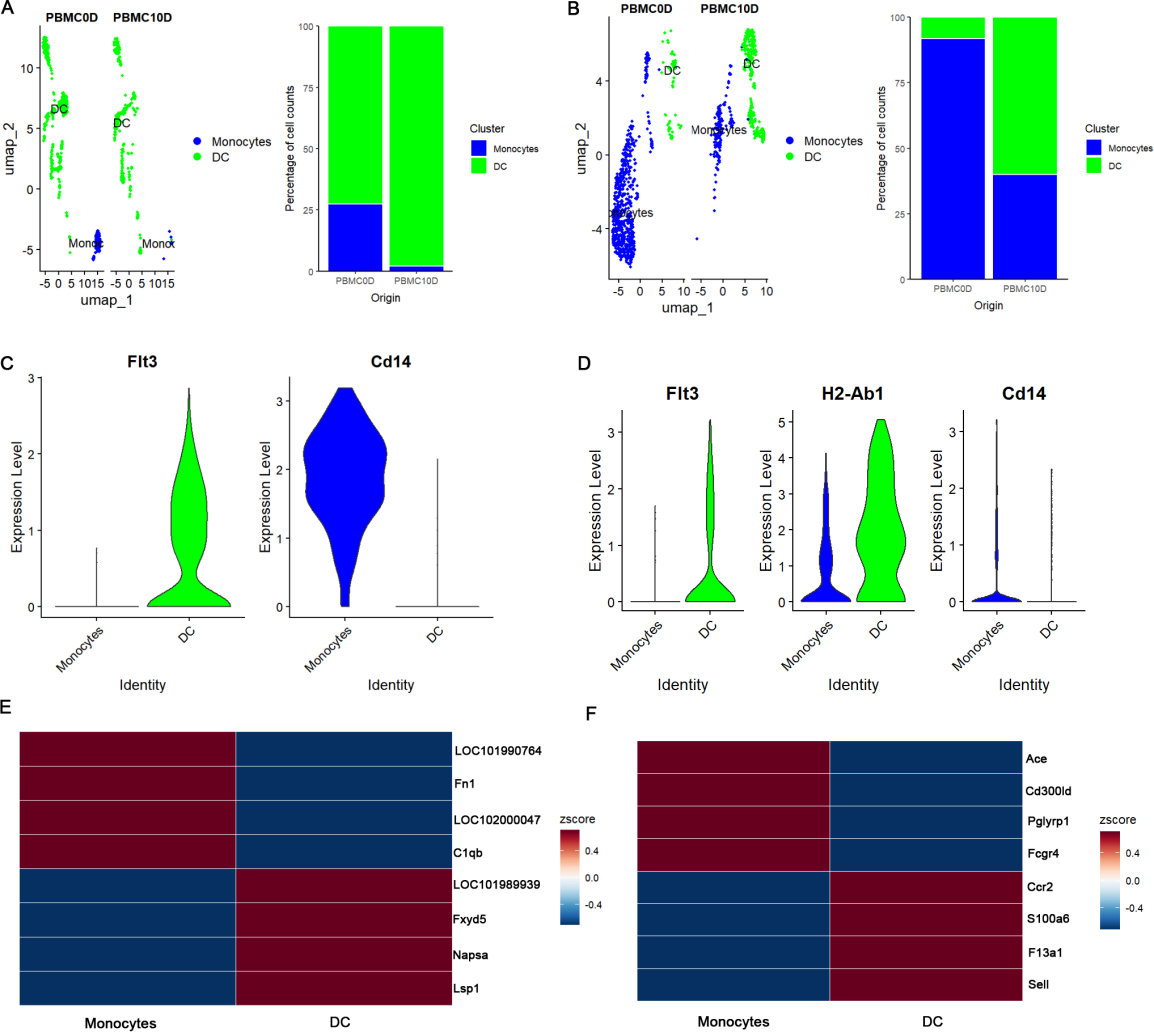
Characterization of MPs. **(A)** UMAP plot and percentage change of MP subsets across conditions in *M. fortis*. **(B)** UMAP plot and percentage change of MP subsets across conditions in Kunming mice. **(C)** Expression patterns of Flt3 and Cd14 in *M. fortis*. **(D)** Expression patterns of Flt3 and Cd14 in Kunming mice. **(E)** Variable feature in *M. fortis* monocyte and DC. **(F)** Variable feature in Kunming mice monocyte and DC.

Next, we considered the MPs to dissect their heterogeneity. Based on the conserved markers, we divided the MPs into monocytes, characterized by the expression of Cd14, and DCs, characterized by the expression of Flt3 (**Figures 2A–D**). To identify additional markers, we generated a heatmap displaying the top differentially expressed genes between monocytes and DC in the two species. We found that Fn1 and C1qb were highly expressed in *M. fortis* monocytes, while Kunming mice monocytes highly expressed Ace and Fcgr4. Similarly, *M. fortis* DC highly expressed Fxyd5, Lsp1, and Napsa, while the DC from Kunming mice highly expressed Ccr2, S100a6, F13a1, and Sell (**Figures 2E, F**). By comparing the percentage change of the MPs before and after infection, we observed that the percentage of DCs increased after infection, but that of monocytes decreased in both *M. fortis* and Kunming mice (**Figures 2A, B**). However, there were more DCs in the PBMCs of *M. fortis* when compared to Kunming mice, while the number of monocytes was more elevated in Kunming mice (**Figures 2A, B**).

We further performed DEG analysis in monocytes and DC to identify the genes responding to the invading worm. Interestingly, we found that the Ccr2 was upregulated in both *M. fortis* and Kunming mice monocytes (**Supplementary Files 1**, **2**), but Cxcl9 was only detected as DEG in *M. fortis* (**Supplementary File 1**). Regarding DC, we observed that infection induced the upregulation of Irf7 and Fcgrt in *M. fortis* DC (**Supplementary File 3**), but not in Kunming mice, suggesting that the two species exhibit distinct responses to the invading worms (**Supplementary File 4**).

### 3.3 T cell characterization

**Figure 3:**
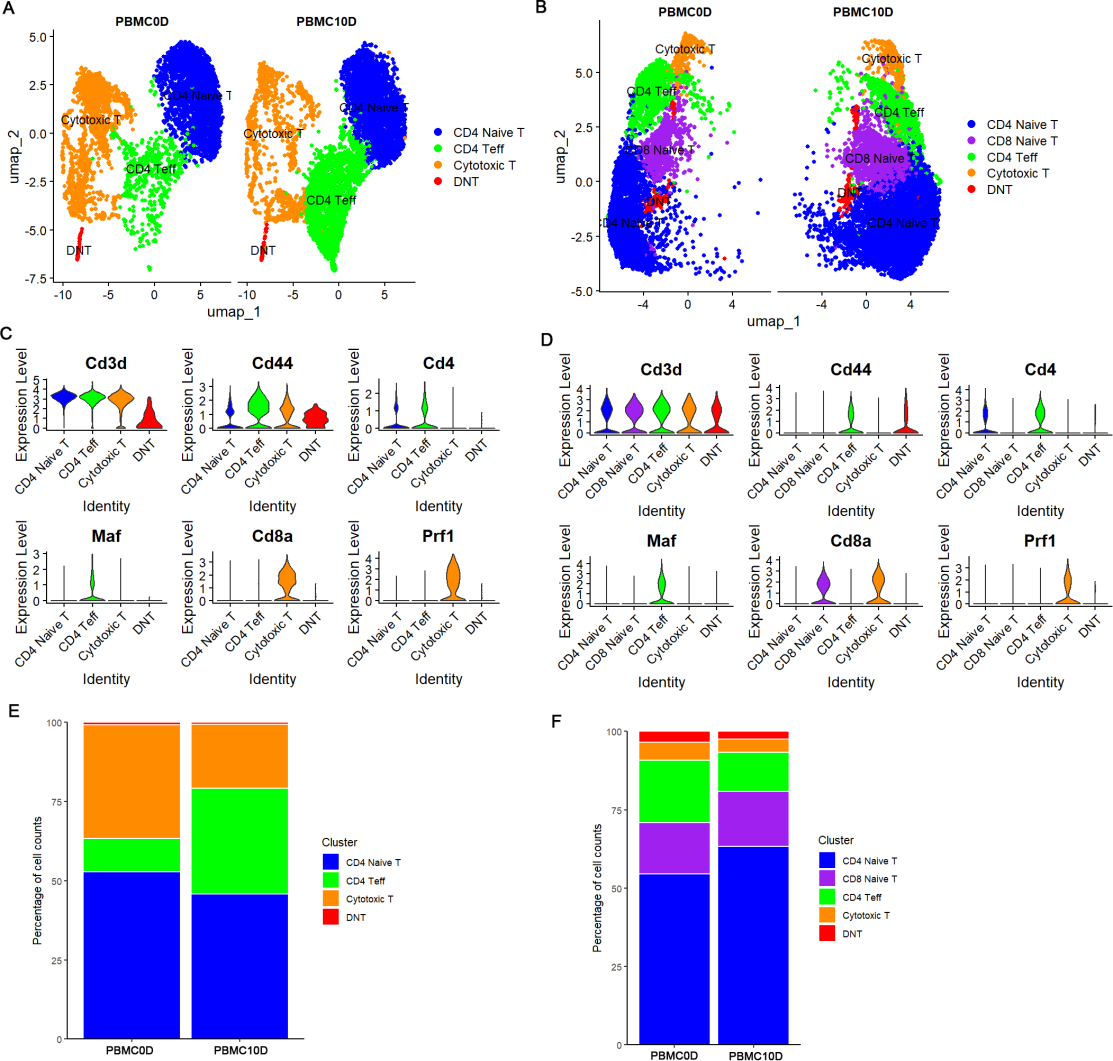
T cells characterization. **(A)** UMAP plot of *M. fortis* T cell subsets annotation cluster. **(B)** UMAP plot of Kunming mice T cell subsets annotation cluster. **(C)** Violin plot of the marker genes used to distinguish *M. fortis* T cell subsets. **(D)** Violin plot of the marker genes used to distinguish Kunming mice T cell subsets. **(E)** Percentage change of *M. fortis* T cell subsets. **(F)** Percentage change of Kunming mice T cell subsets.

We further performed unsupervised clustering on T cell data to identify the T cell subsets present in PBMCs of the two species. Based on the expression of Cd3d, Cd4, Cd8a, Cd44, Maf and Prf1, we grouped the T cells from *M. fortis* into four T cell subsets, including CD4 naive T cells, CD4 effector T (CD4 Teff) cells characterized by the specific expression of Maf, cytotoxic T cells characterized by the expression of Cd8a and Prf1, and double negative T (DNT) characterized by the lack of Cd4 and Cd8a markers but expressing Cd3d (**Figures 3A, C**). Similarly, we grouped Kunming mice T cells into 5 subsets, including those identified in *M. fortis* and CD8 naive T cells expressing Cd8a but not expressing the effector molecule Prf1 (**Figures 3B, D**). By comparing the T cell subpopulations across conditions, we observed that the percentage of CD4+ T effector cells increased in *M. fortis* at 10 dpi, but not in Kunming mice. In contrast, the percentage of cytotoxic T cells decreased in both *M. fortis* and Kunming mice (**Figures 3E, F**).

**Figure 4:**
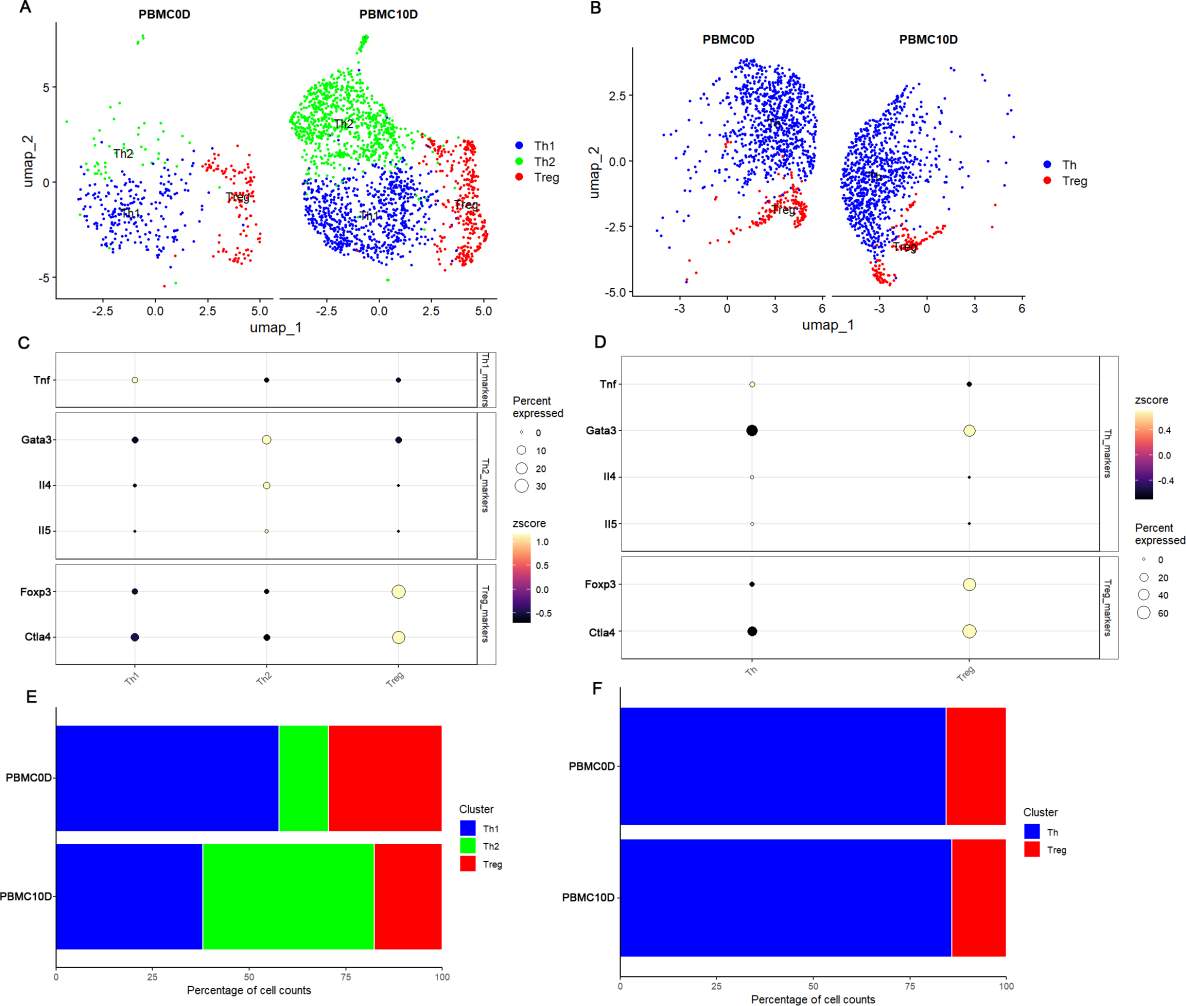
Characterization of CD4 Teff. **(A)** UMAP plot of *M. fortis* CD4 Teff subsets. **(B)** UMAP plot of Kunming mice CD4 Teff subsets. **(C)** Expression patterns of the marker genes used to distinguish the CD4 Teff subsets in *M. fortis*. **(D)** Expression patterns of the marker genes used to distinguish the CD4 Teff subsets in Kunming mice. **(E)** Percentage change of *M. fortis* CD4 Teff subsets. **(F)** Percentage change of Kunming mice CD4 Teff subsets.

We further considered the CD4 Teff cells to decipher their heterogeneity. Based on the UMAP plot and marker genes, we identified 3 distinct clusters in *M. fortis*: a cluster with high expression of Tnf that we annotated as Th1 cells, a cluster highly expressing Gata3, IL4, and IL5 that we annotated as Th2, and a cluster highly expressing Foxp3 and Ctla4 that we annotated as Treg (**Figures 4A, C, E**). By performing similar analysis in Kunming mice, we identified 2 stable clusters: a cluster with a relatively high expression of Tnf, IL4 and IL5, but low expression of Gata3 that we annotated as Th cells because the unsupervised clustering and marker genes failed to distinguish the Th cell subsets, and a cluster with high expression of Foxp3 and Ctla4 that we annotated as Treg (**Figures 4B, D, F**). By comparing the cell type percentages before and after infection, we observed that the percentage of Th2 cells increased, while that of Th1 cells decreased after infection.

We further merged the data from clusters expressing CD4 markers and those expressing CD8a markers to perform a DEGs analysis across conditions, providing a better understanding of the transcriptome profiles of CD4 and CD8 T cells. Interestingly, we found that IL2 and IL4 were upregulated in *M. fortis* CD4 T cells (**Supplementary File 5**), while they were not detected as DEGs in Kunming mice (**Supplementary File 6**). In addition, we observed that Ccr2 and Tcf7 were upregulated in *M. fortis* CD8 T cells (**Supplementary File 5**), but not detected as DEG in Kunming mice CD8 T cells (**Supplementary File 6**).

### 3.4 B cell subsets and responses

**Figure 5:**
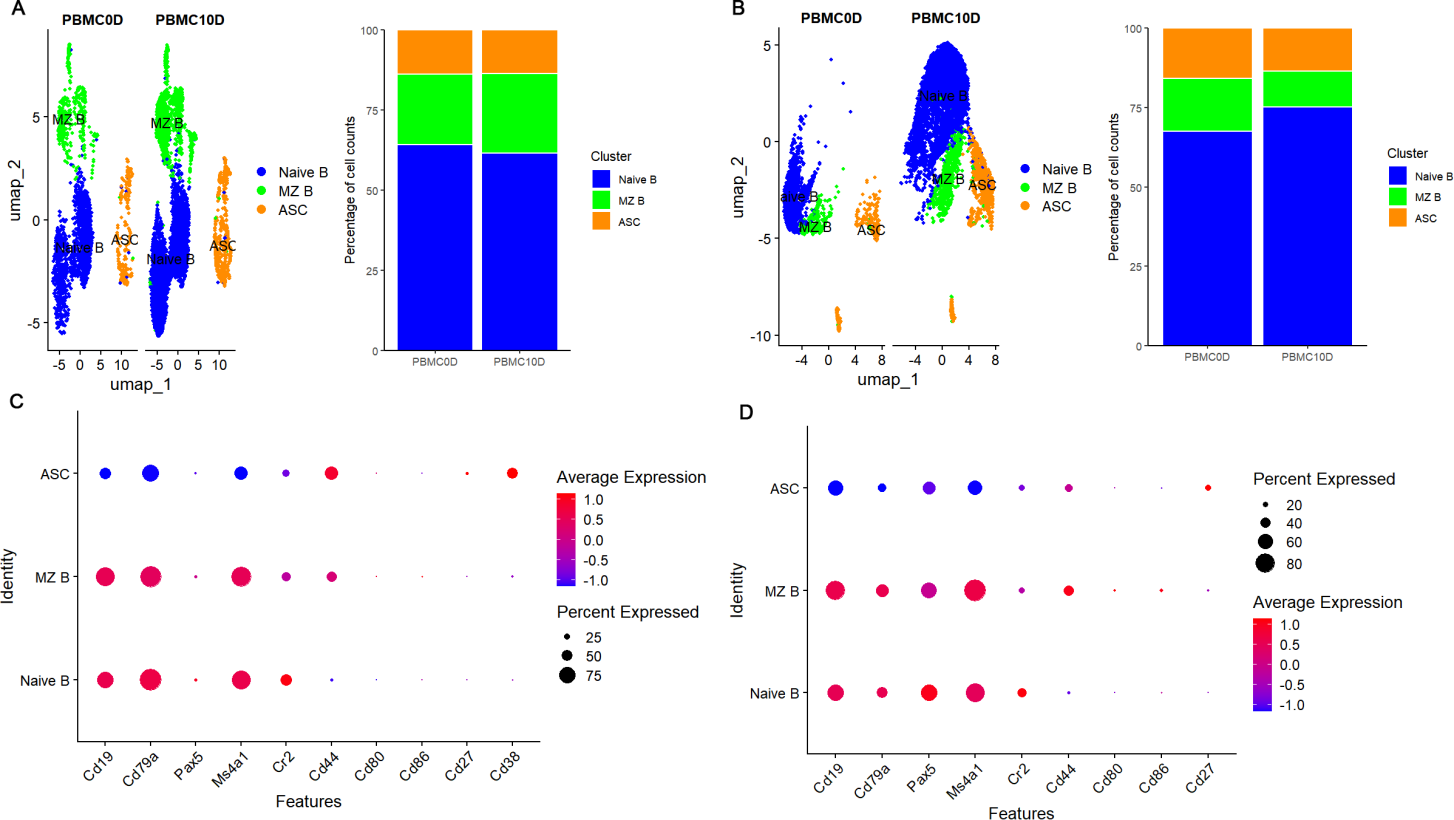
Characterization of B cells. **(A)** UMAP plot and percentage change of B cell subsets across conditions in *M. fortis*. **(B)** UMAP plot and percentage change of B cell subsets across conditions in Kunming mice. **(C)** Expression patterns of the marker genes used to distinguish *M. fortis* B cell subsets. **(D)** Expression patterns of the marker genes used to distinguish Kunming mice B cell subsets.

To determine whether the humoral immune response was significantly activated at 10 dpi, we characterized B cells in the two species. Based on the marker genes, we grouped the B cells into three subsets in both *M. fortis* and Kunming mice. These include naive B cells characterized by the high expression of Cd19, Cd79a, Pax5, Ms4a1 and Cr2, marginal zone (MZ) B cells characterized by the high expression of Cd44, Cd80 and Cd86 when compared to naive B cells, and antibody secreting cells (ASC) characterized by the high expression of Cd27 and Cd38 and decreased expression of Cd19, Cd79a, Pax5 and Ms4a1 (**Figures 5A–D**). By comparing the cell type percentage changes across conditions, we did not observe considerable alteration among the B cell subsets in *M. fortis*; however, the total number and percentage of naive B cells increased in Kunming mice at 10 dpi (**Figures 5A, B**).

**Figure 6:**
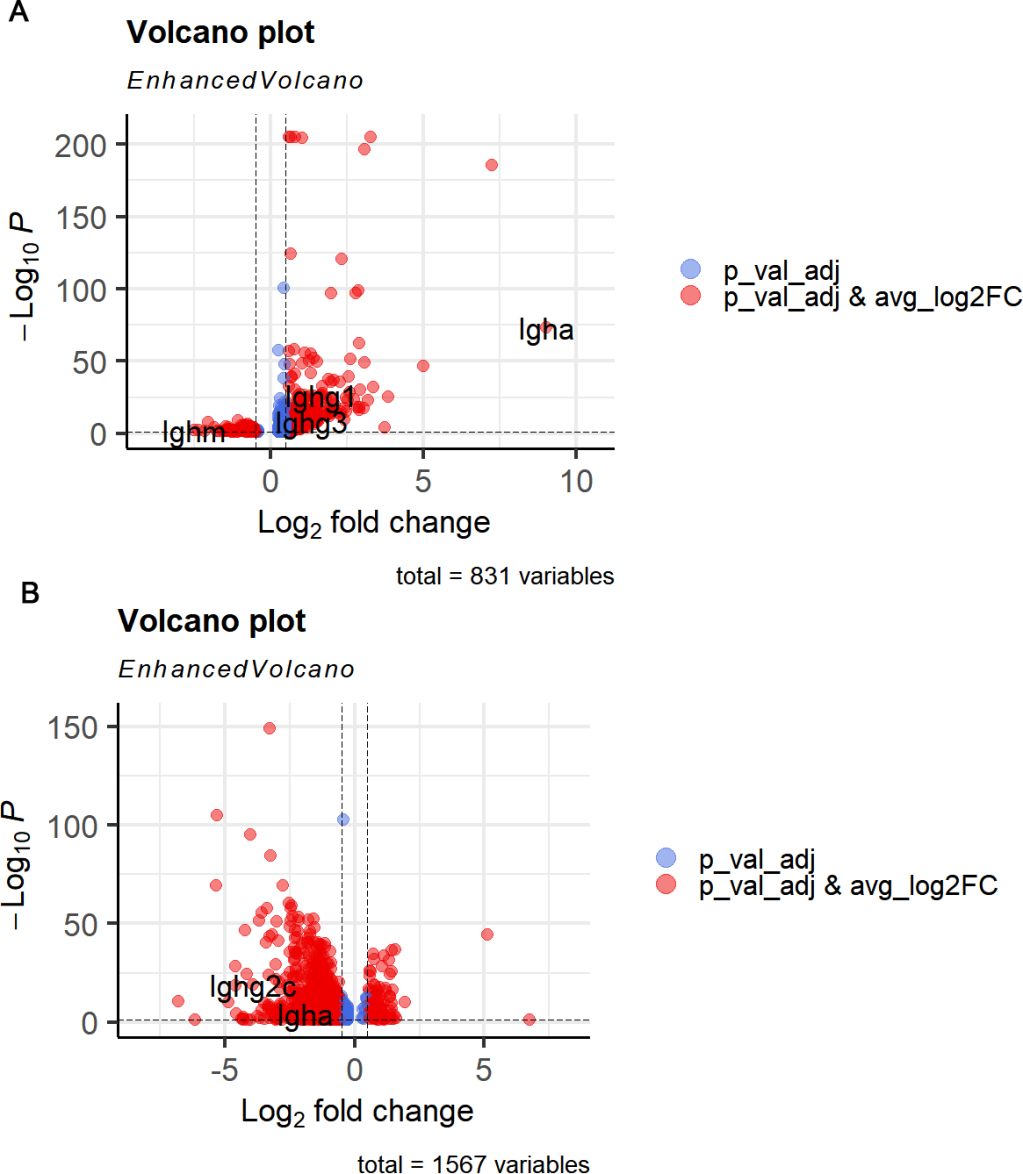
Expression pattern of immunoglobulin genes in the ASCs. **(A)***M. fortis* ASC. **(B)** Kunming mice ASC.

As ASCs are the terminally differentiated B cells that play the key role in humoral immunity, we further screened the DEGs across conditions in this B cell subset. We found that the immunoglobulin genes Igha, Ighg1, and Ighg3 were upregulated in *M. fortis* at 10 dpi, while IghM was downregulated (**Figure 6A**). However, those detected in Kunming mice, including Igha, Ighg2c, were downregulated (**Figure 6B**).

### 3.5 Validation of the expression patterns of some key genes

**Figure 7:**
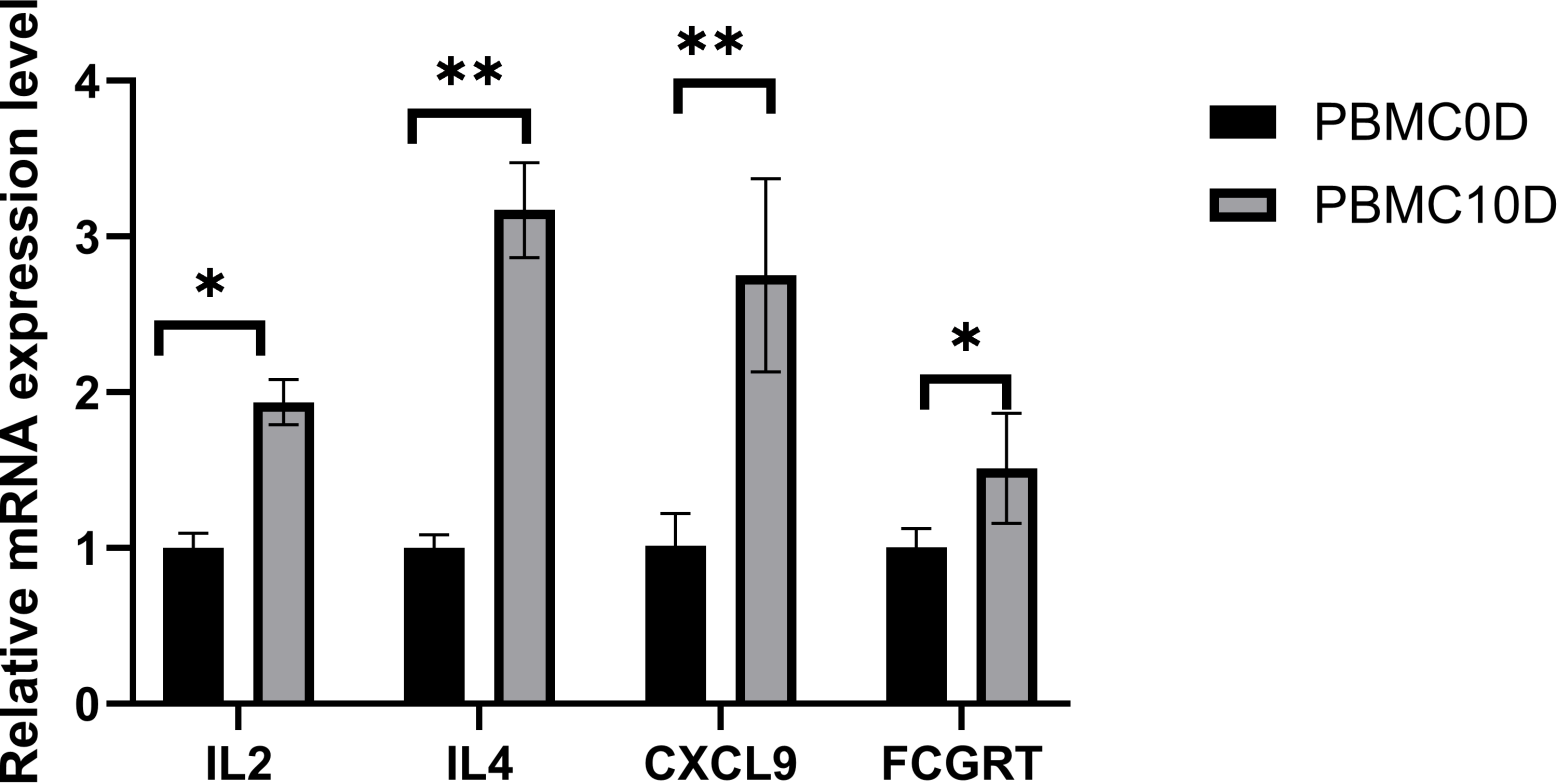
Relative expression of some potential candidate genes selected to validate the scRNA-seq analysis. *P < 0.05, **P < 0.01.

Due to the lack of *M. fortis*-specific antibodies to perform flow cytometry, we selected 4 potential candidate genes, including IL2, IL4, Cxcl9, and Fcgrt, and examined their expression patterns by qPCR in *M. fortis* to validate our scRNA-seq analysis. The qPCR results demonstrated that the expression level of the selected genes increased significantly after infection, suggesting the reliability of the scRNA-seq data (**Figure 7**).

## 4 Discussion

Although bulk RNA sequencing provided some fundamental insights into *M. fortis* immune system, the specific mechanism of its natural resistance to schistosome infection remains unknown (Hu et al., 2014b) (Li et al., 2020). In this study, we have performed single-cell RNA sequencing to characterize and compare the transcriptomic landscape of *M. fortis* and Kunming mice PBMCs at 10 dpi. Based on the conserved marker genes, we identified 6 major cell types in the peripheral blood of the two species, including monocytes, DCs, T cells, NK cells, B cells, and erythrocytes.

By characterizing the MPs, we observed a significant depletion of monocytes in the PBMCs of the infected animals, which may be closely related to the recruitment and adhesion of large numbers of monocytes to the sites of infection, where they can differentiate into macrophages to combat the invading worms (Chen et al., 2022) (Girgis et al., 2014). In addition, we found that Cxcl9 was significantly upregulated in *M. fortis*. As a pro-inflammatory chemokine responsible for the recruitment of Th1 cells and macrophages to the site of infection, Cxcl9 may play an important role in the mechanism of *M. fortis* resistance (Tian et al., 2022) (Hasegawa et al., 2021) (Barron and Wynn, 2011). A previous study reported that a large number of macrophages adhered to the surface of *S. japonicum* from the second week post-infection in *M. fortis*, contributing to worm killing through trogocytosis (Shen et al., 2023). However, it has been reported that *Schistosoma* antigens decrease Cxcl9 production in human PBMCs during human T cell lymphotropic virus type 1 (HTLV-1) infection, suggesting that the migrating schistosomula can downregulate Cxcl9 in the permissive host to prevent immune attack (Lima et al., 2017).

Dendritic cells act as immune sentinels and are essential for capturing the worm’s antigens and initiating the adaptive immunity (Houlder et al., 2023) (Mickael et al., 2025). Our scRNA-seq data suggest that the population of DC increased in the peripheral blood of both *M. fortis* and Kunming mice after infection. This increase indicates the systemic mobilization of the immune system to deal with the migrating schistosomula (Peng et al., 2022). However, Fcgrt and Irf7 were only upregulated in *M. fortis*. Previous studies have also reported that Irf7 was upregulated in *M. fortis* liver at 10 dpi (Li et al., 2020). Although the role of Irf7 has not been elucidated in schistosomiasis, it is well established that Irf7 contributes to host defense against viral and bacterial infection by stimulating the production of the type I interferon (IFN-I), thereby promoting an inflammatory response (Qing and Liu, 2023). Fcgrt code for the neonatal Fc receptor (FcRn), a molecule essential for extending the life of immunoglobulin G (IgG) and facilitating their transport, suggesting that Fcgrt upregulation could promote the humoral immunity against the migrating schistosomula (Roopenian and Akilesh, 2007) (Baker et al., 2013).

Regarding T and B lymphocytes, we found that IL2 and IL4 were significantly upregulated in *M. fortis* CD4+ T cells. Previous studies have also reported that the level of IL2 and IL4 was much higher in the serum of *M. fortis* than in C57BL/6 mice after infection with *S. japonicum* (Hu et al., 2017). IL2 acts as a T cell growth factor, while IL4 drives Th2 cells differentiation (Tatsumi and Kumamoto, 2025) (Allen and Sutherland, 2014). This Th2 response is essential for B cell activation, resulting in antibody production that contributes to worm killing by triggering complement activation and antibody-dependent cell-mediated cytotoxicity, leading to the formation of membrane attack complexes (Shen et al., 2020) (Torben et al., 2012). Our scRNA-seq data revealed that the genes encoding IgA, IgG1, and IgG3 were specifically upregulated in *M. fortis* ASCs, which supports the above-mentioned statement (Li et al., 2020). However, it has been reported that the natural level of IgG3 is much higher in the serum of *M. fortis* than in the serum of mice, indicating that the production of the aforementioned antibodies may be due to the natural background activity of *M. fortis’s* immune system (Jiang et al., 2008). In addition, we found that Tcf7, which modulates the differentiation and function of mature CD8+ T cells, was significantly upregulated in *M. fortis* (Zhang et al., 2021). Tcf7 may also promote the differentiation of naïve CD4+ T cells into Th2, suggesting that Tcf7 can play an important role in the mechanism of *M. fortis* resistance (Zhu et al., 2015).

This study analyzed the overall immune response of two species with different susceptibilities to schistosomiasis using scRNA-seq of the PBMCs. The data generated in the study provide a valuable comparative cellular atlas, revealing differences in immune gene expression and cellular population dynamics between the two species. However, the study has many shortcomings. The main limitation is the sample size resulting from budget constraints, which limits the statistical power of the study. In addition, the raw read data of *M. fortis* were mapped to a mouse reference genome, and our analysis focuses solely on 10 dpi, but lacks earlier or later time points and a cellular atlas from the lung and liver, where the invading worms migrate. These limitations lead to underestimation of gene counts and reduced power to detect weakly expressed genes, making it difficult to capture the full immune dynamics. Also, the lack of traditional immunochemical validation assays presents a methodological limitation of this study. However, qPCR provided indirect validation by detecting the expression of some key genes. For further studies, we recommend investigating lung and liver immune responses using scRNA-seq and flow cytometry with a large sample size. This approach will help better understand the immune response at the site of infection and target the cells of interest for a follow-up study.

## 5 Conclusion

Our study provided the PBMCs’ immune landscape of *M. fortis* during the early phase of infection. We found that the expression level of Cxcl9 increased significantly in *M. fortis* after infection. In addition, we observed that the population of CD4+ effector T cells and the expression level of IL2 and IL4 increased specifically in *M. fortis* after infection. However, further study is needed to validate our findings using a large sample size, which could increase the statistical power. We plan to develop *M. fortis*-specific antibodies in the future and investigate the immune cell signature in the lung and liver of *M. fortis* by combining scRNA-seq and flow cytometry, which will allow us to draw a stronger conclusion.

## Data Availability

The datasets presented in this study can be found in online repositories. The names of the repository/repositories and accession number(s) can be found below: https://www.ncbi.nlm.nih.gov/geo/, GSE308662.

## References

[B40] Allen J. E. Sutherland T. E. (2014). Host protective roles of type 2 immunity: parasite killing and tissue repair, flip sides of the same coin. Semin. Immunol. 26, 329–340. doi: 10.1016/j.smim.2014.06.003, PMID: 25028340 PMC4179909

[B3] Amaral M. S. Santos D. W. Pereira A. S. A. Tahira A. C. Malvezzi J. V. M. Miyasato P. A. . (2021). Rhesus macaques self-curing from a schistosome infection can display complete immunity to challenge. Nat. Commun. 12, 6181. doi: 10.1038/s41467-021-26497-0, PMID: 34702841 PMC8548296

[B37] Baker K. Rath T. Flak M. B. Arthur J. C. Chen Z. Glickman J. N. . (2013). Neonatal fc receptor expression in dendritic cells mediates protective immunity against colorectal cancer. Immunity 39, 1095–1107. doi: 10.1016/j.immuni.2013.11.003, PMID: 24290911 PMC3902970

[B29] Barron L. Wynn T. A. (2011). Macrophage activation governs schistosomiasis-induced inflammation and fibrosis. Eur. J. Immunol. 41, 2509–2514. doi: 10.1002/eji.201141869, PMID: 21952807 PMC3408543

[B25] Chen F. El-Naccache D. W. Ponessa J. J. Lemenze A. Espinosa V. Wu W. . (2022). Helminth resistance is mediated by differential activation of recruited monocyte-derived alveolar macrophages and arginine depletion. Cell Rep. 38. doi: 10.1016/j.celrep.2021.110215, PMID: 35021079 PMC9403845

[B11] Derbois C. Palomares M.-A. Deleuze J.-F. Cabannes E. Bonnet E. (2023). Single cell transcriptome sequencing of stimulated and frozen human peripheral blood mononuclear cells. Sci. Data. 10, 433. doi: 10.1038/s41597-023-02348-z, PMID: 37414801 PMC10326076

[B18] Dobin A. Davis C. A. Schlesinger F. Drenkow J. Zaleski C. Jha S. . (2013). STAR: ultrafast universal RNA-seq aligner. Bioinformatics 29, 15–21. doi: 10.1093/bioinformatics/bts635, PMID: 23104886 PMC3530905

[B26] Girgis N. M. Gundra U. M. Ward L. N. Cabrera M. Frevert U. Loke P. (2014). Ly6C(high) monocytes become alternatively activated macrophages in schistosome granulomas with help from CD4+ cells. PloS Pathog. 10, e1004080. doi: 10.1371/journal.ppat.1004080, PMID: 24967715 PMC4072804

[B28] Hasegawa T. Venkata Suresh V. Yahata Y. Nakano M. Suzuki S. Suzuki S. . (2021). Inhibition of the CXCL9-CXCR3 axis suppresses the progression of experimental apical periodontitis by blocking macrophage migration and activation. Sci. Rep. 11, 2613. doi: 10.1038/s41598-021-82167-7, PMID: 33510341 PMC7844264

[B13] He Y. Luo X. Yu X. Lin J. Liu S. Zhang X. . (1999a). Preliminary study on the levels of natural antibodies against Schistosoma japonicum in Microtus fortis in Dongting lake area. Zhongguo Ji Sheng Chong Xue Yu Ji Sheng Chong Bing Za Zhi. 17, 132–134., PMID: 12563827

[B6] He Y. Luo X. Zhang X. Yu X. Lin J. Li Y. . (1999b). Immunological characteristics of natural resistance in Microtus fortis to infection with Schistosoma japonicum. Chin. Med. J. (Engl). 112, 649–654., PMID: 11601263

[B5] He Y.-X. Salafsky B. Ramaswamy K. (2001). Host–parasite relationships of Schistosoma japonicum in mammalian hosts. Trends Parasitology. 17, 320–324. doi: 10.1016/S1471-4922(01)01904-3, PMID: 11423374

[B10] Hertaeg J. Salazar U. Vom Berg J. LeibundGut-Landmann S. Oehm A. W. Schnyder M. (2025). *In vitro* cytokine response of circulating mononuclear cells from healthy dogs to stage-specific antigens of Angiostrongylus vasorum. BMC Vet. Res. 21, 548. doi: 10.1186/s12917-025-04977-5, PMID: 41039483 PMC12492945

[B32] Houlder E. L. Costain A. H. Nambuya I. Brown S. L. Koopman J. P. R. Langenberg M. C. C. . (2023). Pulmonary inflammation promoted by type-2 dendritic cells is a feature of human and murine schistosomiasis. Nat. Commun. 14, 1863. doi: 10.1038/s41467-023-37502-z, PMID: 37012228 PMC10070318

[B14] Hu Y. Lu W. Shen Y. Xu Y. Yuan Z. Zhang C. . (2012). Immune changes of Schistosoma japonicum infections in various rodent disease models. Exp. Parasitol. 131, 180–189. doi: 10.1016/j.exppara.2012.03.022, PMID: 22521591

[B38] Hu Y. Sun L. Yuan Z. Xu Y. Cao J. (2017). High throughput data analyses of the immune characteristics of Microtus fortis infected with Schistosoma japonicum. Sci. Rep. 7, 11311. doi: 10.1038/s41598-017-11532-2, PMID: 28900150 PMC5595801

[B15] Hu Y. Xu Y. Lu W. Quan H. Shen Y. Yuan Z. . (2014a). Effects of Microtus fortis lymphocytes on Schistosoma japonicum in a bone marrow transplantation model. Exp. Parasitol. 142, 27–37. doi: 10.1016/j.exppara.2014.04.005, PMID: 24746640

[B24] Hu Y. Xu Y. Lu W. Yuan Z. Quan H. Shen Y. . (2014b). *De novo* assembly and transcriptome characterization: novel insights into the natural resistance mechanisms of Microtus fortis against Schistosoma japonicum. BMC Genomics 15, 417. doi: 10.1186/1471-2164-15-417, PMID: 24886088 PMC4073500

[B42] Jiang S.-F. Wei M.-X. Lin J.-J. Pan C.-E. Qiu Q.-W. He Y.-Y. . (2008). Effect of IgG3 antibody purified from sera of Microtus fortis against Schistosoma japonicum. Zhongguo Ji Sheng Chong Xue Yu Ji Sheng Chong Bing Za Zhi. 26, 34–36., PMID: 18637582

[B8] Khan S. Kaihara K. A. (2019). “ Single-cell RNA-sequencing of peripheral blood mononuclear cells with ddSEQ,” in Single cell methods: sequencing and proteomics. Ed. Proserpio V. ( Springer New York, New York, NY), 155–176. doi: 10.1007/978-1-4939-9240-9_10, PMID: 31028637

[B4] Li H. He Y. Y. Lin J. J. (2000). The observation for the phenomenon of Microtus fortis aganisting Schistosoma japonicum. Chin. J. Vet. Parasitol. 8, 12–15.

[B19] Li H. Wang Z. Chai S. Bai X. Ding G. Li Y. . (2020). Genome assembly and transcriptome analysis provide insights into the antischistosome mechanism of Microtus fortis. J. Genet. Genomics 47, 743–755. doi: 10.1016/j.jgg.2020.11.009, PMID: 33753019

[B20] Liao Y. Smyth G. K. Shi W. (2014). featureCounts: an efficient general purpose program for assigning sequence reads to genomic features. Bioinformatics 30, 923–930. doi: 10.1093/bioinformatics/btt656, PMID: 24227677

[B31] Lima L. M. Cardoso L. S. Santos S. B. Oliveira R. R. Oliveira S. C. Góes A. M. . (2017). Schistosoma antigens downregulate CXCL9 production by PBMC of HTLV-1-infected individuals. Acta Tropica. 167, 157–162. doi: 10.1016/j.actatropica.2016.12.030, PMID: 28040482

[B1] LoVerde P. T. (2019). Schistosomiasis. Adv. Exp. Med. Biol. 1154, 45–70. doi: 10.1007/978-3-030-18616-6_3, PMID: 31297759

[B21] Mangiola S. Doyle M. A. Papenfuss A. T. (2021). Interfacing Seurat with the R tidy universe. Bioinformatics 37, 4100–4107. doi: 10.1093/bioinformatics/btab404, PMID: 34028547 PMC9502154

[B17] Martin M. (2011). Cutadapt removes adapter sequences from high-throughput sequencing reads. EMBnet J. 17, 10. doi: 10.14806/ej.17.1.200

[B33] Mickael C. Kumar R. Fonseca Balladares D. C. Nolan K. Lee M. H. Kassa B. . (2025). Role of conventional dendritic cells in schistosomiasis-induced pulmonary hypertension. Clin. Science. 139, 1187–1198. doi: 10.1042/CS20256896, PMID: 41055564 PMC12687420

[B2] Molehin A. J. McManus D. P. You H. (2022). Vaccines for human schistosomiasis: recent progress, new developments and future prospects. IJMS 23, 2255. doi: 10.3390/ijms23042255, PMID: 35216369 PMC8879820

[B34] Peng J. Federman H. G. Hernandez C. M. Siracusa M. C. (2022). Communication is key: Innate immune cells regulate host protection to helminths. Front. Immunol. 13. doi: 10.3389/fimmu.2022.995432, PMID: 36225918 PMC9548658

[B35] Qing F. Liu Z. (2023). Interferon regulatory factor 7 in inflammation, cancer and infection. Front. Immunol. 14. doi: 10.3389/fimmu.2023.1190841, PMID: 37251373 PMC10213216

[B23] Rio D. C. Ares M. Hannon G. J. Nilsen T. W. (2010). Purification of RNA using TRIzol (TRI reagent). Cold Spring Harb. Protoc. 2010. doi: 10.1101/pdb.prot5439, PMID: 20516177

[B36] Roopenian D. C. Akilesh S. (2007). FcRn: the neonatal Fc receptor comes of age. Nat. Rev. Immunol. 7, 715–725. doi: 10.1038/nri2155, PMID: 17703228

[B7] Shen J. Xiang S. Peng M. Zhou Z. Wu Z. (2020). Mechanisms of Resistance to Schistosoma japonicum Infection in Microtus fortis, the Natural Non-permissive Host. Front. Microbiol. 11. doi: 10.3389/fmicb.2020.02092, PMID: 33013763 PMC7494751

[B30] Shen J. Zhao S. Peng M. Li Y. Zhang L. Li X. . (2023). Macrophage-mediated trogocytosis contributes to destroying human schistosomes in a non-susceptible rodent host, Microtus fortis. Cell Discov. 9, 101. doi: 10.1038/s41421-023-00603-6, PMID: 37794085 PMC10550985

[B39] Tatsumi N. Kumamoto Y. (2025). The role of IL-2 in type 2 immunity. Front. Immunol. 16. doi: 10.3389/fimmu.2025.1622187, PMID: 41000383 PMC12457388

[B27] Tian Y. Wen C. Zhang Z. Liu Y. Li F. Zhao Q. . (2022). CXCL9-modified CAR T cells improve immune cell infiltration and antitumor efficacy. Cancer Immunol. Immunother. 71, 2663–2675. doi: 10.1007/s00262-022-03193-6, PMID: 35352167 PMC10991162

[B41] Torben W. Ahmad G. Zhang W. Nash S. Le L. Karmakar S. . (2012). Role of antibody dependent cell mediated cytotoxicity (ADCC) in Sm-p80-mediated protection against Schistosoma mansoni. Vaccine 30, 6753–6758. doi: 10.1016/j.vaccine.2012.09.026, PMID: 23000221 PMC3488153

[B16] Wang S. Sun S.-T. Zhang X.-Y. Ding H.-R. Yuan Y. He J.-J. . (2023). The evolution of single-cell RNA sequencing technology and application: progress and perspectives. Int. J. Mol. Sci. 24, 2943. doi: 10.3390/ijms24032943, PMID: 36769267 PMC9918030

[B9] Wang Z. Wu L. Qian H. Fu Y. Chen T. Du L. . (2025). PBMCs of Hainan Black goats mediating immune response to resist the infection of Mannheimia haemolytica. Microb. Pathog. 210, 108183. doi: 10.1016/j.micpath.2025.108183, PMID: 41232700

[B43] Zhang J. Lyu T. Cao Y. Feng H. (2021). Role of TCF-1 in differentiation, exhaustion, and memory of CD8^+^ T cells: A review. FASEB J. 35, e21549. doi: 10.1096/fj.202002566R, PMID: 33913198

[B22] Zhang Y. Li B. Duan J. Chen X. Zhang X. Ye J. . (2023). SynEcoSys: a multifunctional platform of large-scale single-cell omics data analysis. doi: 10.1101/2023.02.14.528566

[B44] Zhu Y. Wang W. Wang X. (2015). Roles of transcriptional factor 7 in production of inflammatory factors for lung diseases. J. Transl. Med. 13, 273. doi: 10.1186/s12967-015-0617-7, PMID: 26289446 PMC4543455

[B12] Zilionis R. Engblom C. Pfirschke C. Savova V. Zemmour D. Saatcioglu H. D. . (2019). Single-cell transcriptomics of human and mouse lung cancers reveals conserved myeloid populations across individuals and species. Immunity 50, 1317–1334.e10. doi: 10.1016/j.immuni.2019.03.009, PMID: 30979687 PMC6620049

